# Testing the modifiability of episodic future thinking and episodic memory among suicidal and nonsuicidal adolescents

**DOI:** 10.1002/jcv2.12236

**Published:** 2024-04-08

**Authors:** Pauline Goger, Rachel J. Nam, Nathan Lowry, Kerri‐Anne Bell, Neha Parvez, Olivia H. Pollak, Donald J. Robinaugh, Daniel L. Schacter, Christine B. Cha

**Affiliations:** ^1^ Department of Counseling and Clinical Psychology Teachers College Columbia University New York New York USA; ^2^ Department of Psychology Northeastern University Boston Massachusetts USA; ^3^ Department of Psychology Harvard University Cambridge Massachusetts USA

**Keywords:** adolescents, cognition, episodic future thinking, episodic memory, suicide

## Abstract

**Background:**

Despite increased attention on treatment and prevention for suicidal adolescents, we know little about potential intervention targets. Episodic future thinking—the ability to imagine detailed, personal, and future‐oriented events—is a modifiable cognitive process that has been linked with suicidal ideation (SI) in adolescents. However, until now its modifiability has only been tested in adults.

**Method:**

Adolescents (*N* = 176, ages 15–19; 71% SI) completed performance‐based measures of episodic future thinking (i.e., Experimental Recombination Paradigm) and memory immediately before and after an Episodic Specificity Induction (ESI).

**Results:**

Adolescents produced a greater number of future episodic details after (vs. before) the ESI but showed no change in non‐episodic details (e.g., semantic information). Patterns of change in episodic future thinking were not moderated by SI history. Adolescents overall did not demonstrate change in past episodic detail counts after the ESI. However, there were select moderating effects of SI history on this effect.

**Conclusion:**

Results show that episodic future thinking can change immediately following an episodic specificity induction among adolescents, regardless of whether they have previously experienced SI. This demonstration of within‐person change constitutes a foundational first step in examining malleability of episodic future thinking in adolescents and offers preliminary evidence of a cognitive mechanism that may be leveraged in service of reducing adolescents' SI.

**Key points**

**What's known?**

- Episodic future thinking—the ability to imagine detailed, personal, and future‐oriented events—is a cognitive/process that has been linked with suicidal ideation (SI) in adolescents. However, until now its modifiability has only been tested in adults.

**What's new?**

- Episodic future thinking is also modifiable in adolescents, as adolescents were able to produce significantly more episodic details after a single brief specificity induction.

**What's relevant?**

- Findings can be used as a foundation to further examine the episodic future thinking construct as a potential target for intervention for suicidal adolescents.

## INTRODUCTION

Although suicidal thoughts and behaviors (i.e., STBs) are a concern across the lifespan (CDC, 2022), adolescence is an especially high‐risk period. Adolescents typically navigate new roles and challenges (Christie & Viner, 2005), which exacerbate the risk of developing both psychopathology associated with STBs (e.g., depression, PTSD; Low et al., 2012) and STBs specifically (Bilsen, 2018). Indeed, in 2020 over 2000 youth (15–19 years) in the United States alone died by suicide, representing the third leading cause of death for this age group (CDC, 2021, 2022). Rates for STBs have also been steadily increasing (Curtin & Heron, 2019) and are quite alarming: the 2019 US Youth Risk Behavior Survey found that nearly 1 in 5 high school students seriously considered attempting suicide, nearly 1 in 7 made a plan, and close to 1 in 10 attempted suicide (Ivey‐Stephenson et al., 2020).

Given this increased risk and the fact that STBs not only tend to recur for a higher‐risk subset of adolescents (Goldston et al., 2016; van Vuuren et al., 2021) but are associated with pervasive adverse outcomes in adulthood (Reinherz et al., 2006), adolescence may be an especially opportune time for intervention to alleviate current and future functional impairment and distress. Unfortunately, only a fraction of STB risk research focuses on youth (Franklin et al., 2017) and current treatment approaches lack desired effectiveness. Although some treatments, such as dialectical behavior therapy (DBT), cognitive behavioral therapy (CBT), and brief interventions during high risk periods do show promise (Cha et al., 2018; Glenn et al., 2019), meta‐analyses have found that treatment efficacy is low and has not improved over the last 50 years overall (Harris et al., 2022). These findings indicate that new intervention programs may be needed and that identifying novel malleable treatment targets to build treatments from the ground up may improve future intervention efficacy and effectiveness (NIMH Strategic Plan, 2015).

### Episodic future thinking and episodic memory in adults

One potentially promising treatment target is episodic future thinking, or the ability to imagine^1^ a specific personal event occurring in the future (Schacter et al., 2017). As this cognitive process is associated with problem solving abilities and divergent creative thinking (Jing et al., 2016; Madore et al., 2014, 2015; Madore, Szpunar, et al., 2016), suicide theories consider future thinking to be a protective factor that may reduce one's sense of entrapment and ultimately impede the development or worsening of STBs by enabling one to consider alternative scenarios in times of distress (O’Connor, 2011; Williams et al., 1996). Indeed, suicidal adults, compared to nonsuicidal counterparts, have been shown to display future thinking deficits, such as using fewer future‐tense verbs in speech as well as having greater difficulty imagining temporally specific and positive future events (Cha et al., 2022; Greaves, 1971; Williams et al., 1996).

Similarly, episodic memory (i.e., the ability to recall a specific personal event from the past; Tulving, 1983), which is closely related to episodic future thinking (Schacter & Addis, 2007), has also evidenced impairment in suicidal adults. In particular, suicidal adults tend to exhibit overgeneral episodic memory, which can result in negative outcomes such as prolonged affective disturbances, impaired problem solving skills, and limited specificity of future thinking (Pollock & Williams, 2001; Williams et al., 1996, 2006). Most promising to potential future suicide interventions, both episodic future thinking and episodic memory have been shown to be malleable via specificity inductions that promote construction of more detailed mental scenes or events in both healthy (Hallford et al., 2022; Jing et al., 2016; McFarland et al., 2017; Madore et al., 2014; Madore, Szpunar, et al., 2016; Madore & Schacter, 2016; for review, see Schacter & Madore, 2016) and clinically depressed (McFarland et al., 2017) adults. Improvements in these cognitive constructs have been associated with positive outcomes on clinically relevant measures in nonclinical populations, such as reduced symptoms of anxiety (Jing et al., 2016), and studies testing a more extensive intervention targeting memory specificity in clinical populations have also shown improvements in depression (Hallford et al., 2021; Neshat‐Doost et al., 2013; Raes et al., 2009) and posttraumatic stress disorder (Moradi et al., 2014).

### Episodic future thinking and episodic memory in adolescents

Although most of the extant literature on these cognitive processes pertains to adult samples, preliminary findings in adolescents point to episodic future thinking and its malleability as a promising avenue to explore in this younger age group. As youth age from childhood through adolescence, substantial development of future thinking abilities takes place (Gott & Lah, 2014; Steinberg et al., 2009), with any disruption or slowing of this developmental process being linked with adverse outcomes and appropriate development with positive ones. For example, faster future orientation development has been associated with faster improvement in hopelessness in adolescents (Mac Giollabhui et al., 2018), and the ability to think about the future is related to feeling a closer connection to one's future self (McCue et al., 2019). Greater episodic future thinking abilities have also been associated with diminished impulsivity in the form of lesser delay discounting, which is the degree to which someone disregards greater future rewards in favor of smaller, more proximal rewards (Bromberg et al., 2015). This lower impulsivity, in turn, is associated with lower likelihood of engaging in harmful behaviors, such as attempting suicide (Dawes et al., 2008), using substances (Moeller & Dougherty, 2002), or overeating (Loxton, 2018). In fact, behavioral health interventions have successfully leveraged these associations to address obesity in youth (Sze et al., 2015) and binge‐drinking behavior in college students (Voss et al., 2022), for example, by asking participants to repeatedly and vividly imagine positive future events they are looking forward to. Importantly, however, while these interventions encourage engaging in positive episodic future thinking more frequently, no research thus far has examined the *malleability* of future thinking itself in suicidal adolescents.

Recently, Cha et al. (under review) reported the first study to utilize a performance‐based episodic future thinking task in adolescents, a procedure known as the Experimental Recombination Paradigm (ERP; Addis et al., 2009), which requires participants to imagine novel future events based on person, object, and location details that are recombined from participants' episodic memories. Cha et al. (under review) found that the ability to imagine specific actions tied to an imagined future event is associated with both history and future likelihood of suicidal ideation (SI). The same study demonstrated that episodic future thinking and episodic memory are associated with one another during this developmental period.

The current study aimed to build on this link between episodic future thinking and SI in adolescents by examining whether episodic future thinking and episodic memory processes are malleable in adolescents, which is a foundational first step in determining whether this construct should be more thoroughly probed in the future. First, we aimed to test whether episodic detail counts produced when adolescents imagined future events would increase after the Episodic Specificity Induction (ESI). Based on previous studies that used the same specificity induction in adults (e.g., Madore et al., 2014; for review, see Schacter & Madore, 2016), we expected an increase in episodic detail counts but no change in non‐episodic detail counts when imagining future events. We also aimed to ensure that any changes following the ESI do not simply reflect general changes in narrative style, which is the way in which individuals describe a scene or tell a story. Accordingly, we included a picture description task previously used by Gaesser et al. (2011) and Madore et al. (2014) in studies of young and older adults. Based on the findings of Madore et al. (2014), we also expected no change in general narrative style as assessed by this task. Second, we aimed to examine whether episodic future thinking malleability differs based on SI group status. Finally, we tested whether participants would also report more detailed episodic memories after the ESI, as has been found in previous studies of young and old adults (Madore et al., 2014; Madore & Schacter, 2016).

## METHOD

### Participants

The Institutional Review Board at Teachers College, Columbia University approved all study procedures. Participants were adolescents (*N* = 176; ages 15–19; *M*_age_/*SD* = 17.60/1.33; 69.3% female at birth) who were recruited from the community in the greater New York metropolitan area. Participants were excluded if they (1) were unable to effectively participate in the study, (2) reported imminent suicidal intent, (3) presented with violent or very agitated behavior, (4) were not fluent in English, or (5) were under 18 years of age *and* did not have a guardian or caregiver able to participate. The initial sample consisted of 180 adolescents, but two withdrew from the study during the baseline assessment and an additional two were excluded due to lacking ERP data (more detail below). The recruitment strategy included aiming for equal distribution between those participants who did and did not experience SI within the past year. However, this was based on the initial phone screen, and participants who subsequently reported SI at the baseline appointment were retained, but included in the SI group, leading to an enriched sample of SI participants.

### Experimental design

**FIGURE 1:**
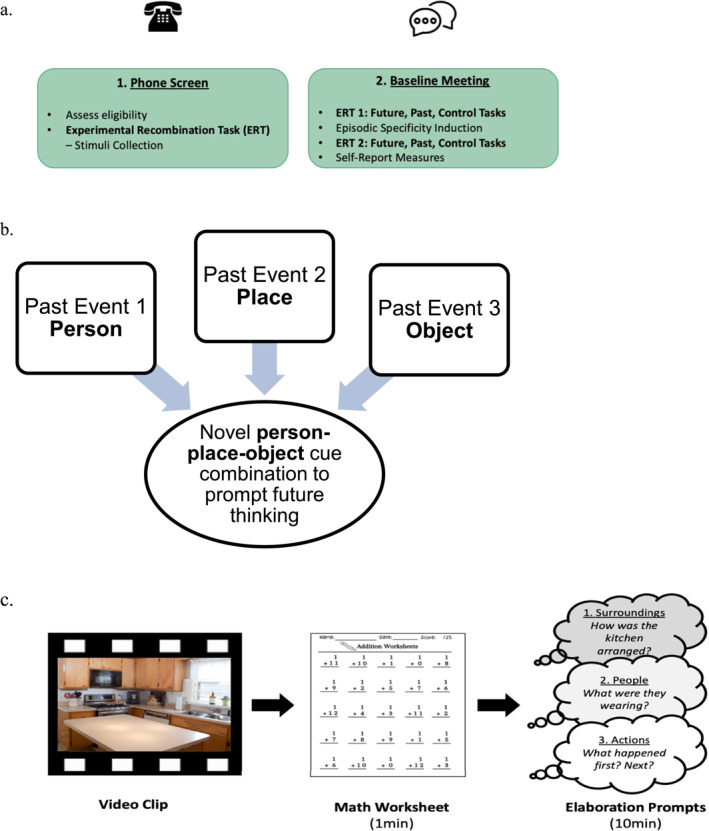
Study procedures and task detail depictions. (A) Study procedures. (B) Future thinking cue construction from experimental recombination task (Addis et al., 2009). (C) Specificity induction, adapted from Madore et al. (2014); Schacter and Madore (2016). Only aspects of the study relevant to the current paper scope are featured here. ERT cues were counterbalanced but remained in the same order from ERT1 to ERT2. In 1a, steps are listed in order of administration from top to bottom within each box.

Participants completed this study in three parts: (1) an initial eligibility phone screen, (2) an in‐person (81.2%) or virtual (18.8% due to Covid‐19) laboratory visit, and (3) an online follow up assessment. Eligible adolescents completed the first portion of the ERP (stimulus collection, described below) during the initial phone screen (see Figure 1A). The preliminary stimulus collection phase occurred approximately 1–3 weeks before the baseline visit. During the baseline visit, adolescents and their guardians completed questionnaires and adolescents then completed the Picture Description Task, the second portion of the ERP, and the ESI. Lastly, email‐initiated follow‐up assessments occurred 3 and 6 months after the laboratory visit. For the baseline assessment adolescents received a $35 and guardians, if applicable, a $25 gift card; for the 3‐ and 6‐month follow‐ups adolescents were entered into $50 and $100 gift card raffles, respectively. In the current study, only data from the baseline visit are used, as the ERP and ESI were only completed at this visit and not follow‐up assessments.

### Measures and procedure

#### Picture description task (PDT)

During the PDT (Gaesser et al., 2011) participants provided 3 min descriptions of complex images (e.g., photographed nature scene) which were coded into episodic and semantic details to assess their general narrative style. This task does not rely on prospection or episodic memory and has been shown to remain unaffected by the ESI (Madore et al., 2014). This task was administered once before and once after the ESI during the laboratory visit to ensure that observed group differences were truly due to prospection and episodic memory deficits rather than unintended effects of the ESI.

#### Experimental recombination paradigm (ERP)

The ERP (Addis et al., 2009) is a performance‐based episodic future thinking task that is divided into two parts: a preliminary stimulus collection phase and an event (re‐)generation phase. For the preliminary stimulus collection, adolescents were asked to retrieve personal memories from 10 negative and 10 positive events that were specific, lasted less than 24 h, and occurred in the past 5 years. For each of the 20 events, adolescents provided information about a person, object, and location associated with the event and supplied a brief event title. This information was then used to (1) create past event cue slides by randomly selecting 2 positive past event and 2 negative past event descriptions in their entirety and (2) create future event cue slides by re‐combining person, object, and location cues randomly selected from the 20 events into event cues for 2 positive and 2 negative novel future events (see Figure 1B).

The second part of the ERP took place during the baseline visit. Once before and once after ESI, adolescents were presented with one: (1) past positive, (2) past negative, (3) future positive, and (4) future negative computerized cue slide in addition to the (5) control task. These were presented in counterbalanced (i.e., on valence) but blocked (i.e., on tense) order to minimize burden of switching back‐and‐forth between past and future cues. Either of the five cues could be randomly presented first, such that past, future, or control cues were presented first, in the middle, or last, depending on the participant. The order before the ESI remained the same after the induction. Participants were prompted to provide as many spatiotemporal and perceptual details as possible in 3 min for each of the events and were asked to imagine future events in as plausible a way as possible. These descriptions were audio‐recorded, and the recordings subsequently coded by rigorously trained (i.e., needing an ICC = 0.75–1.00 in order to independently code) research assistants unaware of participants' group status (i.e., SI vs. no‐SI group) to identify the number of episodic and non‐episodic details per event (Levine et al., 2002).

Episodic details are also referred to as *internal details* (IDs) and pertain to specific people or situations. These were further divided into five subtypes: (1) *events* (ID‐Event; i.e., physical occurrences and actions occurring around or toward the adolescent), (2) *place* (ID‐Place; i.e., spatial localization of the adolescent), (3) *time* (ID‐Time; i.e., temporal localization of the adolescent), (4) *perception* (ID‐Perceptual; i.e., sensory experiences of the adolescent), and (5) *emotion/thought* (ID‐Emotion/Thought; mental state of the adolescent). Non‐episodic details are also referred to as *external details* (EDs), which include semantic details (i.e., factual knowledge about the world), general commentary, and any information provided about other events.

#### Episodic specificity induction (ESI)

The ESI is adapted from the Cognitive Interview, a forensic protocol used to enhance eyewitnesses' accurate recall of experienced events (Fisher & Geiselman, 1992; Memon et al., 2010). It has been shown to boost the number of internal details that people provide during episodic future thinking and episodic memory tasks (Jing et al., 2016; Madore et al., 2014; Madore, Jing, et al., 2016; Madore & Schacter, 2014; Madore, Szpunar, et al., 2016; McFarland et al., 2017; Sheldon et al., 2019). In the standard procedure, participants first view a brief video, and are then given a math worksheet filler task for about 1 min, followed by prompts to retrieve episodic details from the video. They then perform memory, future thinking, and other tasks. In this study, participants viewed a brief video of a routine event or scene (e.g., adults completing mundane tasks in the kitchen), after which they were (a) told that they are the chief expert about the video; (b) instructed to recreate a mental image of the surroundings, people, and actions in the video; and (c) probed about details of each (e.g., location of objects in the room; what people were wearing; sequence of actions). The ESI procedure lasted approximately 10 min (see Figure 1C).

#### Demographic and clinical assessments

Adolescents and their guardians completed a demographics questionnaire including questions about age, sex, ethnicity, gender, sexual orientation, and socioeconomic status.

SI and other self‐injurious thoughts and behaviors were assessed with the *Self‐Injurious Thoughts and Behaviors Interview*—*Revised* (SITBI‐R; Fox, Harris, et al., 2020; Gratch et al., 2022). The SITBI‐R is a structured interview assessing SI, suicide plan, suicide attempts, and non‐suicidal self‐injury. In the current paper, participants were classified as belonging to the SI group versus non‐SI group if they either endorsed a gateway question from the SITBI‐R (i.e., ‘Have you ever had *thoughts* of killing yourself?’) or checked off one of eight items describing passive or active suicidal thoughts (e.g., ‘I wish I were dead,’ ‘I should kill myself’).

### Data analytic plan

Data were examined for completeness and distribution before hypothesis testing. One participant was identified as a meaningful outlier in analyses containing narrative style and baseline SI symptoms and was therefore excluded from analyses examining these factors. Out of 1408 total ERP trials, we excluded 24 trials (1.70%). The most common reasons for exclusion were that the participant did not follow the provided task instructions (*n* = 11; e.g., talked about a past event older than 5 years), the participant did not recognize the provided stimuli (*n* = 7; e.g., did not recognize cue(s) or did not remember a past event), and there were administrative errors (*n* = 4). We also excluded trials where the participant was interrupted by an external event during the task (*n* = 1) and the participant withdrew mid‐task (*n* = 1). Given that missingness in the ERP data was quite low (∼5%), analyses were completed with raw data. We used *α* = 0.05 per analysis as this research area is relatively new and results are intended to stimulate future hypothesis‐generation as well as advance current questions.

#### Aim 1: Malleability of episodic future thinking

Paired samples *t*‐tests were used to examine changes of each ERP count (i.e., total ID counts and all subtypes, ED counts) and the control narrative style task from before to after the ESI. For total ID and ED counts we examined both positively and negatively valenced events regardless of the outcomes of the overall (i.e., average across positive and negative events) counts, in order to more fully understand whether changes after the ESI may be dependent on event valence. For subtypes we took a stepwise approach: (1) subtypes were only examined if the total ID count significantly differed from pre‐to post‐ESI, (2) subtypes were only broken down into positively and negatively valenced events if the overall subtypes exhibited a significant change from pre‐to post‐ESI.

#### Aim 2: Suicidal‐group status and change in episodic future thinking

Mixed models were used to examine interactions between ERP change from before to after the ESI and SI‐group status. SI‐group effect was a fixed effect, the intercept a random effect, and time (i.e., before and after induction) was a continuous repeated measures effect. When an interaction was significant, estimated marginal means were examined in order to qualify the nature of the interaction. As in Aim 1, total ID counts were further divided into positively and negatively valenced events, while subtypes were examined on a step‐wise basis.

#### Aim 3: Malleability of episodic memory

The same statistical approaches from Aims 1 and 2 were applied in order to examine episodic memory.

## RESULTS

The final overall sample was diverse with 57.1% of adolescents identifying as belonging to a racial minoritized group. Approximately 70% of participating adolescents reported recent SI (i.e., within the past year; SI group; *n* = 126), while the remainder denied recent or lifetime history of ideation or attempt (i.e., no‐SI group; *n* = 50). Youth in the SI group were more likely to be female at birth, bisexual, Hispanic, depressed, and anxious, and less likely to be cis‐gender or heterosexual (see Table 1).

**TABLE 1:** Baseline sample characteristics.

	Total (*N* = 176)	SI group (*N* = 126)	No‐SI group (*N* = 51)	ES
Age, *M* (SD)	17.60 (1.33)	17.69	17.38	−0.24
Sex (% female)	69.3	79.8	57.4	0.23*
Gender identity (% cis‐gender)	87.0	83.1	97.7	0.19*
Sexual orientation				
Heterosexual	54.0	45.5	81.6	0.34**
Homosexual	8.5	9.9	6.1	
Bisexual	25.6	33.1	10.2	
Ethnicity (% non‐Hispanic)	78.1	74.2	87.8	−0.15
Race				
White	42.9	38.8	53.1	0.25*
Asian	22.4	19.8	28.6	
Black	16.5	17.4	14.3	
Other	15.3	19.8	4.1	
Unknown	2.9	4.1	0.0	
Parent college graduate	70.4	67.8	77.3	0.09
Depression (QIDS‐SR)	8.27 (5.12)	9.88 (4.98)	4.19 (2.90)	−1.27**
Anxiety (SCARED)	31.14 (16.57)	36.59 (15.48)	17.90 (10.67)	−1.31**

*Note*: Effect sizes represent Cohen's *d* values for baseline group comparisons of continuous variables, and phi values for baseline group comparisons of categorical variables.

Abbreviations: ES, effect size; *M*, mean; *SD*, standard deviation.

*p < 0.05, **p < 0.001.

### Aim 1: Malleability of episodic future thinking

Adolescents' episodic future thinking abilities (i.e., ID counts) improved over time, such that adolescents produced more total overall IDs after (vs. before) the ESI (*t* = −2.54, *d* = 0.20, *p* = 0.012; see Supplemental Figure S1).^2^ This pattern of improvement maintained across IDs pertaining to both positive and negative future thoughts (*t* = −2.46–2.02, *ds* = 0.16–0.20, *ps* = 0.015–0.045). Specific types of IDs, however, did not evidence a statistically significant increase (*t*s = −1.94–1.15, *d*s = 0.09–0.16, *p*s = 0.054–0.251). See Supplemental Figure S1.

As expected, adolescents' non‐episodic future thinking abilities (i.e., ED counts) did not significantly change after (vs. before) the ESI. This was the case for overall, positive, and negative ED counts (*t*s = 0.47–0.96, *d*s = 0.04–0.08, *p*s = 0.339–0.639). Similarly, adolescents did not produce more episodic details on the control task (i.e., PDT ID counts) following the ESI (*t* = −1.61, *d* = 0.13, *p* = 0.109), although there was a slight increase after removal of an outlier (*t* = −1.98, *d* = 0.15, *p* = 0.049). Nonetheless, we did not observe a three‐way interaction between time (pre‐vs. post ESI), task (ERP vs. PDT), and detail type (ID vs. ED; *F*_1,153_ = 0.001, *p* = 0.973).

### Aim 2: Suicidal‐group status and change in episodic future thinking

**FIGURE 2:**
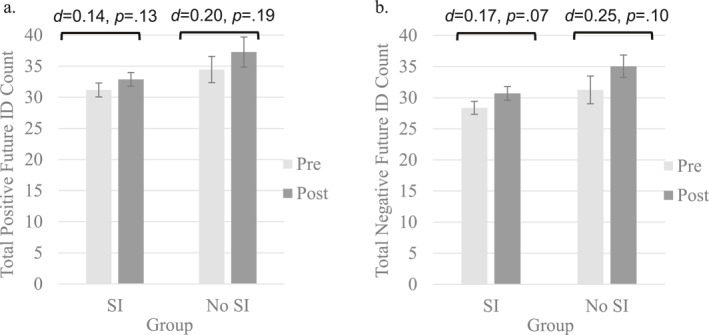
Episodic future thinking detail counts before and after specificity induction by baseline SI‐group status. (A) Positive future event detail counts. (B) Negative future event detail counts. Episodic future thinking represented by counts of ID (internal details) generated for future events. Detail counts captured via number of internal details (IDs). Pre and Post refer to before and after the specificity induction. Error bars represent standard errors.

Adolescents' episodic future thinking abilities as captured via overall ID as well as positive and negative IDs did not change as a function of SI group status (*F*s_1,160_ = 0.234–1.318, *p*s = 0.253–0.629). See Figure 2. However, adolescents without SI history produced on average more details after (*M* = 36.40 vs. 31.68, *SD* = 12.55 vs. 10.38, *t* = 2.50, *d* = 0.43, *p* = 0.013), but not before (*M* = 32.17 vs. 30.02, *SD* = 13.59 vs. 10.41, *t* = 1.10, *d* = 0.19, *p* = 0.279) the ESI than adolescents without SI history.

### Aim 3: Malleability of episodic memory

**FIGURE 3:**
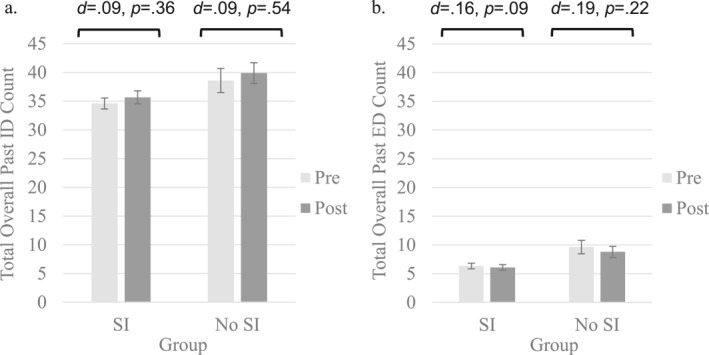
Episodic memory detail counts before and after specificity induction by baseline SI‐group status. (A) Total overall past internal detail counts. (B) Total overall past external detail counts. Episodic memory represented by counts of ID (internal details) generated for past events. Non‐episodic memory represented by counts of total overall ED (external details). Pre and Post refer to before and after the specificity induction. Error bars represent standard errors.

Neither total overall ID counts (*t*s = −1.726 to −0.307, *d*s = 0.02–0.13, *p*s = 0.086–0.759) nor ED counts (*t*s = −0.811–0.143, *d*s = 0.01–0.06, *p*s = 0.419–0.886) differed from before to after the ESI when participants retrieved episodic memories (see Figure 3). Adolescents produced roughly the same number of details after the ESI regardless of the type of detail or event valence. Similarly, mixed models revealed that for episodic memory, response to the ESI did not depend on baseline SI‐group status across any of the overall ID counts (*F*s_1,165_ = 0.027–0.581, *p*s = 0.447–0.870).

## DISCUSSION

The current study is the first to examine the malleability of episodic future thinking in adolescents, laying the groundwork for more thoroughly probing this construct in the context of intervention work in the future. Using an objective measure (i.e., ERP; Addis et al., 2009) to assess future thinking ability, this study evaluated whether episodic detail counts increased after participants completed the ESI (Madore et al., 2014; Schacter & Madore, 2016). Moreover, we examined whether episodic future thinking malleability is moderated by SI history. This investigation yielded three main findings.

First, we observed that adolescents produced more episodic details about future events following the ESI. This finding is consistent with the adult literature, which has shown that episodic future thinking increases immediately following a specificity induction (Hallford et al., 2022; Jing et al., 2016; Madore et al., 2014; Madore & Schacter, 2016; Madore, Szpunar, et al., 2016; McFarland et al., 2017; Sheldon et al., 2019). It appears that prompting adolescents to recall a scene they just observed in order to report on it in a highly detailed way may help them better imagine a future event they are newly constructing. Considering the positive outcomes associated with higher versus lower future thinking ability in adolescents (e.g., lower hopelessness; Mac Giollabhui et al., 2018) its malleability is a promising finding for clinical utility. However, it is unclear whether an increase in future thinking is directly associated with a decrease in SI or—even if unlikely given the generally positive findings in adolescents and adults—it may be associated with an increase in SI. Future studies should probe the prospective association between changes in future thinking and changes in SI. Additionally, in the current study, internal details for both positive and negative future events increased after the ESI, although the clinical utility of this finding also remains to be determined (i.e., it is unclear if increasing the general ability for detailed future thinking may be more, less, or perhaps equivalently beneficial than engaging in valence‐specific future thinking). Many studies utilizing future thinking to change adolescent or young adult behavior ask participants to envision only a positive future event—either one that is already planned or a newly constructed one. It may be that imagining positive future events in greater detail would be associated with well‐being in adolescents because it may increase positive feelings about the future. Alternatively, engaging in positive future thinking, especially in unrealistic positive future thinking, may be unhelpful if the imagined events repeatedly do not occur or perhaps are not as positive as anticipated, making adolescents feel disappointment (Nam & Cha, 2023; Pollack et al., 2021). Similarly, negative future thinking may evoke negative emotions but may also help adolescents engage in proactive problem‐solving or coping, decreasing the likelihood that the event will be as negative as anticipated. However, it is also possible that being more detailed and specific when thinking about the future in general may be helpful in combating avoidance or overly positive or negative expectations regardless of the type of event imagined.

Second, counter to expectations, episodic memory internal detail counts did not increase following the ESI. This finding was somewhat surprising given that episodic future thinking and episodic memory are closely linked (Schacter et al., 2007; Schacter & Madore, 2016), and that adult studies show that episodic memory is malleable, typically showing the same increase in internal details following a specificity induction as observed for future thinking (Hallford et al., 2022; Jing et al., 2016; Madore et al., 2014; Madore & Schacter, 2016; Madore, Szpunar, et al., 2016). It is possible that this discrepancy was a result of different memory tasks and specificity inductions used between studies. For example, in one study (Hallford et al., 2022) participants were prompted to be as detailed and vivid as possible and use mental imagery in direct relation to the event they were asked about. In contrast, in the current study participants experienced a more subtle induction without direct prompting, which may not have extended to past events. Also, previous studies using the same ESI as in the current study have not used the same behavioral task (i.e., ERP); they have instead usually provided word or picture prompts and asked participants to produce less events that are only constrained by time (i.e., future or past) and not by content as in the ERP (i.e., person/place/object details). It is also possible that adolescents experienced a ceiling effect where they already provided more episodic memory details before the induction than adults may have, as adolescents and emerging adults (ages 16–24) tend to perform better across different episodic memory tasks than adults (Pauls et al., 2013). Indeed, when interpreting results and examining whether that could be the case in our study, we found that the past average ID count was higher (*M* = 35.61) than the future average ID count (*M* = 30.81; *p* < 0.001; *d* = 0.40).

Third, the changes from before to after the ESI for episodic future thinking did not vary based on whether participants did or did not have a history of SI. Regardless of SI history, generated event details increased after the ESI, although adolescents without SI history tended to produce significantly more details after the ESI than those with SI history. Previous findings in youth and adults support the outcome that suicidal adolescents may have a deficit in future thinking (Cha et al., under review, 2022), but whether or how the reported details before and after the ESI might change on the basis of suicidality was so far unknown. If the current finding of episodic detail increases is replicated in future studies, it would indicate that those adolescents who may need support from a specificity induction the most would be able to benefit from a boost to their episodic future thinking. A lingering question is whether this approach would work with currently suicidal teens. In the current study, adolescents who endorsed any suicidal thoughts within the last year were grouped as having SI‐history, which would encompass both adolescents with fleeting symptoms about a year ago and those who may have had severe symptoms quite recently. Examining suicidality in a more granular fashion may provide additional insights into the possible effects of the ESI.

### Limitations

Our findings should be interpreted in the context of study limitations. First, we did not utilize a control induction as in previous studies with young and old adults (for review, see Schacter & Madore, 2016), making it difficult to determine if findings from this study are attributable to the ESI or other extraneous variables (e.g., practice effects). Although our study was designed to maximize power to detect whether a change in future thinking can occur among adolescents, especially those with a history of SI, future studies should include a control induction in order to clarify whether the ESI itself is responsible for the observed effects. Relatedly, our sample was racially diverse (57.1% non‐white) but future work with a more socio‐demographically diverse sample (e.g., age, sex, income) may allow for greater generalizability. It is possible that the ESI may be differentially effective cross‐culturally or based on other individual (e.g., age), family (e.g., income), or clinical (e.g., severity) factors. If this were the case, it would have important implications for treatment development and recommendations as for whom this type of treatment may be best suited.

Another limitation is that the ESI and subsequent ERP task were completed in the same visit, meaning that only immediate short‐term changes were examined, but not long‐term changes. It is likely that observed changes fade or weaken as time passes, but how quickly and how completely they disappear is yet to be determined. Future work should attempt to answer this question as well as determine whether repeated specificity inductions would yield a consistent or greater benefit to the ability to generate more future event. When repeated specificity inductions were used in adults previously, lower depressive and PTSD symptoms were seen at 2‐and 3‐month follow‐up timepoints, indicating that this line of inquiry in suicidal adolescents may be promising (Hallford et al., 2021; Moradi et al., 2014; Neshat‐Doost et al., 2013). Additionally, our ESI took place in the context of an ERP task that asked adolescents to recombine components of past events to construct and describe a novel future event. It is possible that the ESI would have different effects if adolescents were instead describing an actual upcoming event, for example, Similarly, although there is emerging work pertaining to mental imagery among suicidal adolescents (e.g., Lawrence et al., 2021) in the present study we did not include a formal assessment of mental imagery capability. Although adolescents were not required to construct a mental image when imagining the future, they were prompted to do so during the ESI and may have also assumed to do so during the ERP task based on the prompt to “imagine” the future in as much detail as possible. Therefore, variation in this ability may be an important moderator of the effects of the ESI.

Finally, although we did examine the effects of the ESI on episodic future thinking and episodic memory in adolescents with and without SI history, this sample is nonetheless a community‐based one. Further studies in clinical populations are warranted to examine whether present results generalize to adolescents with more acute or severe psychopathology.

### Clinical implications

Despite its limitations, this study sheds light on a potential treatment target that can be leveraged to address the worsening suicidal thoughts and behavior crisis in adolescents. Our previous research has shown that baseline production of future event details is associated with future suicidality (Cha et al., under review), but it was unclear whether future event detail production is malleable in adolescents. The current study is a critical first step in beginning to answer this question, showing that detail production significantly increased after a single brief specificity induction. Considering the current scarcity of effective STB interventions, identifying and examining future thinking as a promising treatment target for intervention in adolescents is important and could have wide‐reaching positive effects if implemented successfully in the future.

## AUTHOR CONTRIBUTIONS

**Pauline Goger**: Data curation; formal analysis; methodology; visualization; writing—original draft. **Rachel J. Nam**: Data curation; formal analysis; methodology; visualization; writing—original draft. **Nathan Lowry**: Investigation; writing—original draft. **Kerri‐Anne Bell**: Investigation; project administration; writing—review and editing. **Neha Parvez**: Investigation; project administration; writing—review and editing. **Olivia H. Pollak**: Investigation; project administration; writing—review and editing. **Donald J. Robinaugh**: Investigation; methodology; writing—review and editing. **Daniel L. Schacter**: Investigation; methodology; writing—review and editing. **Christine B. Cha**: Conceptualization; formal analysis; funding acquisition; methodology; project administration; resources; supervision; writing—review and editing.

## CONFLICT OF INTEREST STATEMENT

The authors have declared they have no competing or potential conflicts of interest.

## ETHICAL CONSIDERATIONS

The Institutional Review Board at Teachers College, Columbia University approved all study procedures.

## Supporting information

Figure S1

## Data Availability

The data that support the findings of this study are available from the senior author (Dr. Christine Cha) upon request.

## ENDNOTES

^1^
“Imagine” refers to thinking about the future in some way, similar to “remember” when thinking about the past; it does not require constructing a mental representation, which would be referred to as “simulation” per established research in the field (e.g., Madore et al., 2016; Schacter et al., 2007).

^2^
When using a Bonferroni corrected alpha = 0.016 accounting for analyses for total overall, positive, and negative future thinking details, the total overall ID change remains significant.
