# Supplementary material for: Testing the modifiability of episodic future thinking and episodic memory among suicidal and nonsuicidal adolescents
Source: JCPP Adv. 2024 Apr 8;4(3):e12236. doi: 10.1002/jcv2.12236 (PMC11472815; doi:10.1002/jcv2.12236)
Supplement: Supplementary file 1 — Figure S1 [file JCV2-4-e12236-s001.docx]

**Supplemental Figure 1. Pre- vs. Post-ESI Comparison of Total Overall Episodic/Non-Episodic Future Thinking and Control Task Detail Counts**

-0.42

0.45 (Per), 0.25 (Pl), 0.41 (E/T), -0.12 (T)

1.15

2.19*

2.62

*Note*. Episodic future thinking represented by counts of total overall ID (internal details) and ID subtypes generated for future events. Non-episodic future thinking represented by counts of total overall ED (external details). Episodic details generated in Picture Description Task represented by ID-Control Task. Numbers next to each line represent pre- to post-ESI detail count changes. Per = ID-Perception, Pl = ID-Place, E/T = ID-Emotion/Thought, T = ID-Time.
** p*<.05; ***p<.01; ***p*<.001.
